# Mechanism of BRG1 silencing in primary cancers

**DOI:** 10.18632/oncotarget.10593

**Published:** 2016-07-13

**Authors:** Stefanie B. Marquez, Kenneth Thompson, Li Lu, David Reisman

**Affiliations:** ^1^ Division of Hematology/Oncology, Department of Medicine, University of Florida, Gainesville, Florida, USA; ^2^ Department of Pathology, University of Florida, Gainesville, Florida, USA

**Keywords:** BRG1, SWI/SNF, mutation, epigenetic, cancer

## Abstract

*BRG1* (*SMARCA4*) is a documented tumor suppressor and a key subunit of the SWI/SNF chromatin remodeling complex that is silenced in many cancer types. Studies have shown that *BRG1* is mutated in cancer-derived cell lines, which led to the assertion that *BRG1* is also mutated in primary human tumors. However, the sequencing of BRG1-deficient tumors has revealed a paucity of mutations; hence, the cause of *BRG1* silencing in tumors remains an enigma. We conducted immunohistochemistry (IHC) on a number of tumor microarrays to characterize the frequency of BRG1 loss in different tumor types. We also analyzed BRG1-deficient tumors by sequencing the genomic DNA and the mRNA. We then tested if BRG1 expression could be induced in BRG1-negative cell lines (i.e., that lack mutations in *BRG1*) after the application of several different epigenetic agents, including drugs that inhibit the AKT pathway. We found that a subset of BRG1-negative cell lines also demonstrated aberrant splicing of BRG1, and in at least 30% of BRG1-deficient tumors, BRG1 expression appeared to be suppressed due to aberrant BRG1 splicing. As the majority of BRG1-deficient tumors lack mutations or splicing defects that could drive BRG1 loss of expression, this suggests that other mechanisms underlie BRG1 silencing. To this end, we analyzed 3 BRG1-deficient nonmutated cancer cell lines and found that BRG1 was inducible in these cell lines upon inhibition of the AKT pathway. We show that the loss of BRG1 is associated with the loss of E-cadherin and up-regulation of Vimentin in primary tumors, which explains why BRG1 loss is associated with a poor prognosis in multiple tumor types.

## BACKGROUND

The SWI/SNF complex has been linked to human cancer since the discovery that the SWI/SNF subunit BAF47 (*SNF5, SMARCB1, INI1*) was a bona fide tumor suppressor that underlies the genesis of malignant Rhabdoid tumors [1]. Since this initial discovery in the mid-1990s, two other SWI/SNF subunits have emerged as potential tumor suppressors: Brahma (*BRM*, *SMARCA2*) and Brahma Related Gene 1 (*BRG1*) [2]. BRM and BRG1 are not found in the same SWI/SNF complex, but rather, they are mutually exclusive catalytic subunits that convert ATP into mechanical energy in order to shift the positions of histones within the chromatin [3–5]. This gives transcription factors and other key cellular proteins access to certain regions of the DNA. The SWI/SNF complex does not serve in a single transduction pathway; instead, it fosters the function of many different cellular proteins and pathways [2], and therefore, this complex serves as a catalyst for gene expression. This role has been exemplified by microarray experiments in yeast, which have shown that SWI/SNF regulates 5-7% of the yeast genome [6].

Our early experiments showed that the expression of both the BRG1 and BRM subunits was absent in about 10-20% of lung cancers [7]. Interestingly, since BRG1 and BRM are the main catalytic subunits of the SWI/SNF complex, their mutual silencing assures that this complex is completely inactivated. As SWI/SNF has been shown to be necessary for development, differentiation, cell adhesion, and growth control, the loss of one or both of these subunits would be predicted to impact cancer development [2, 8]. In particular, both BRG1 and BRM are known to bind to Rb and facilitate Rb-mediated growth inhibition [9–11]. A number of labs have shown that in BRG1/BRM-deficient cell lines, Rb fails to inhibit growth, but that the reconstitution of either BRG1 or BRM restores Rb function. Similarly, p53 function and growth control have been tied to the SWI/SNF complex and specifically to BRG1, and as such, are impacted by the inactivation of SWI/SNF [12–16]. In murine models, the heterozygous inactivation of *BRG1* results in the development of mammary tumors, while the homozygous conditional knockout of *BRG1* potentiates the development of several types of tumors [17–19]. However, the lack of overt high tumorigenesis is observed because even when BRG1 and BRM protein expression is missing to some degree, they may be functionally redundant. These two proteins share approximately a 75% amino acid sequence homology and can substitute for one another in certain experimental models [2, 8].

We and others first examined cancer cell lines to gather a basic understanding of how *BRG1* may be silenced in cancer cells. Wong *et al*. was the first to report *BRG1* mutations after his group sequenced 180 cancer cell lines and found that 18 cell lines harbored nonsense or insertion/deletion mutations; however, only 9 cell lines harbored homozygous mutations that would account for the loss of *BRG1* expression [20, 21]. Previous scientific dogma with respect to the mechanism of *BRG1* silencing has therefore been shaped primarily by these findings in cell lines. *BRG1* silencing caused by mutations has gained further support by a number of recent Next Generation Sequencing (NGS) publications that have identified the presence of primarily missense mutations in a variety of tumors [22, 23]. These studies did not analyze *BRG1*-deficient tumors, and they did not determine the percentage of tumors that harbor a given mutation. In lung cancer, NGS has demonstrated that missense mutations and abrogating mutations occur far more infrequently (< 5%) than the frequency of BRG1 loss (by immunohistochemistry (IHC):15-30%) [24–28]. Moreover, Sanger sequencing studies of human cancer have not documented mutations as the major cause of *BRG1* silencing [28]. Moreover, Medina *et al*. sequenced a series of BRG1-deficient lung cancer-derived cell lines and found that a majority of these cell lines harbored various abrogating (loss of expression) mutations [21]. Based on these studies, a number of other studies then sought to analyze if *BRG1* mutations occur in primary BRG1-deficient tumors. However, these studies have found a paucity of *BRG1* mutations, which is in stark contrast to what has been found in BRG1-deficient cell lines. Thus, abrogating *BRG1* mutations appear to contribute to, but cannot fully account for, the loss of BRG1 expression in the majority of cases. Remarkably, some current research papers and reviews have reported that *BRG1* is silenced through mutations and have neglected to mention that *BRG1* is silenced more frequently than mutations occur; such statements leave the reader to infer that mutations are the major mechanism of inactivation [20, 21, 29, 30]. The mechanism of *BRG1* silencing in human tumors would appear to be unresolved and is therefore a provocative issue.

In this paper, we present a summary of our sequencing data of *BRG1* in cell lines, which parallels the data contributed by other investigators. Uniquely, we uncovered that splicing defects within BRG1 indicate an as yet unidentified mechanism that might be responsible for the silencing of *BRG1* in primary tumors. As *BRG1* has previously been demonstrated to be silenced in a cadre of tumors, we advance the general understanding of the role of BRG1 in cancer by showing that, according to IHC, *BRG1* is silenced in a spectrum of tumor types. In addition to the aberrant splicing of BRG1, we also show that activation of the AKT pathway silences *BRG1*, as AKT pathway inhibitors were found to readily induce BRG1 protein expression. These data give new insights into *BRG1* is altered during cancer progression.

## RESULTS

### In BRG1-deficient primary human cancers, *BRG1* is infrequently silenced by mutations

In order to determine how *BRG1* is silenced in human cancer, we stained a variety of lung and other cancer types. Of these cancers, 30 tumors including 10 lung tumors, were found to be BRG1-deficient by IHC [7]. We obtained genomic DNA from these 30 tumors, and using primer sets that flanked each BRG1 exon, we amplified the exons by PCR and then sequenced all 37 exons from these BRG1-deficient tumors (Supplementary Table 1A). We found no indels, missense or nonsense mutations in any of these tumors, which is consistent with results that were recently reported by Oike *et al*. [31] and Rodriquez-Nieto *et al*. [30]. These investigators sequenced 16 and 12 BRG1-deficient primary lung tumors, respectively, and found 0/16 and 1/12 abrogating mutations that might explain how *BRG1* is silenced in these tumors. The observed rate of abrogating mutations in these two latter studies (3.57%) is similar to the abrogating (nonsense mutations, insertion/deletions) mutation rate in NSCLC as observed in the Atlas (The Cancer Genome Atlas, TCGA) and COSMIC (Catalogue of Somatic Mutations in Cancer) databases (4.6% and 2.2%, respectively) (Table 1) [28].

**Table 1:** Mutations in *BRG1* do not account for its frequency of loss

*SMARCA4* (*BRG1*)	Tumor	TCGA: Percentage			Tumor	Cosmic: Percentage			IHC	IHC	
Mutations	No#	Nabr	Abr	Total		Nabr	Abr	Total	Current Study	Other Studies	References
Acute Myeloid Leukemia	197	0.00%	0.00%	0.00%							
Adrenocortical Carcinoma	80	3.75%	0.00%	3.75%							
Bladder Urothelial Carcinoma	237	5.49%	0.42%	5.91%	Bladder	5.66%	0.00%	5.66%	12.0%		
Brain Lower Grade Glioma	289	3.11%	0.00%	3.11%							
Breast invasive carcinoma	981	0.71%	0.71%	1.43%	Breast	0.78%	0.39%	1.17%	32.0%	52.4%	Bai 2013
Cervical Cancer	39	2.56%	0.00%	2.56%					12.5-75%	2-21% (SCC-ACC)	Kuo 2006
Colon adenocarcinoma	269	0.00%	0.00%	0.00%	Colon	5.75%	1.28%	7.03%	48.0%	8.0%	Watanabe 2011
Endometrial Cancer	248	9.68%	0.00%	9.68%	Endometrium	9.61%	0.36%	9.96%			
Esophageal Cancer	282	2.10%	1.10%	3.20%	Esophageal	4.62%	0.60%	5.23%	3.0%		
Glioblastoma multiforme	291	0.69%	0.00%	0.69%					50.0%	18.4%	Bai 2012
Head/Neck Cancer	306	5.56%	0.33%	5.88%					20.0%		
Kidney Chromophobe	66	1.52%	0.00%	1.52%							
Kidney renal clear cell carcinoma	417	1.68%	0.24%	1.92%	Kidney	1.05%	0.00%	1.05%	55.0%		
Kidney renal papillary cell carcinoma	112	5.36%	0.00%	5.36%							
Lung adenocarcinoma	544	6.07%	3.31%	9.38%	Lung	4.21%	3.06%	7.27%	15-30%	12-15%	Matsubara 2013; Oike 2013
Lung squamous cell carcinoma	178	3.93%	0.56%	4.49%							
Ovarian Carcinoma (Serous)	230	1.74%	0.00%	1.74%	Ovarian	1.61%	0.00%	1.61%	19.0%		
					Serous	1.20%	0.10%	1.30%			
					Clear cell	9.10%	0.00%	9.10%			
Pancreatic adenocarcinoma	57	6.59%	1.10%	7.69%	Pancreatic	1.00%	1.00%	1.99%	12.0%	50.0%	Numata 2013
Prostate adenocarcinoma	251	0.00%	0.00%	0.00%	Prostate	0.0%	0.6%	0.0%	10.0%	67% (weak), 19%	Sun 2007; Li 2006
Rectum adenocarcinoma	116	3.45%	0.00%	3.45%							
Skin Cutaneous Melanoma	345	7.54%	0.29%	7.83%	Melanoma	7.55%	0.63%	8.18%	10.0%	27.0%	Lin 2010
Stomach adenocarcinoma	245	3.27%	0.82%	4.08%	Stomach	1.54%	0.00%	1.54%	0.0%	0.0%	Yamamichi 2007
Thyroid carcinoma	405	0.99%	0.00%	0.99%					8.0%		
Uterine Carcinosarcoma	114	0.88%	0.88%	1.75%							
Hepatocellular Carcinoma	202	6.44%	0.99%	7.43%	Liver	1.63%	0.61%	2.24%	60.0%	65.0%	Endo 2013
Unweighted Average		3.32%	0.43%	3.75%		3.69%	0.58%	4.22%			

We examined and tabulated the mutation rates in 25 and 15 different tumor types from the Cancer Genome Atlas and the Catalogue of Somatic Mutations in Cancer (COSMIC) databases respectively. The number of total tumors analyzed for each tumor type is given followed by the percentages of nonabrogating, abrogating and total mutations for each of the databases. In the following columns, the IHC data obtained in the current study is compared with IHC data in the published literature.

### *BRG1* silencing in human tumors

**Figure 1:**
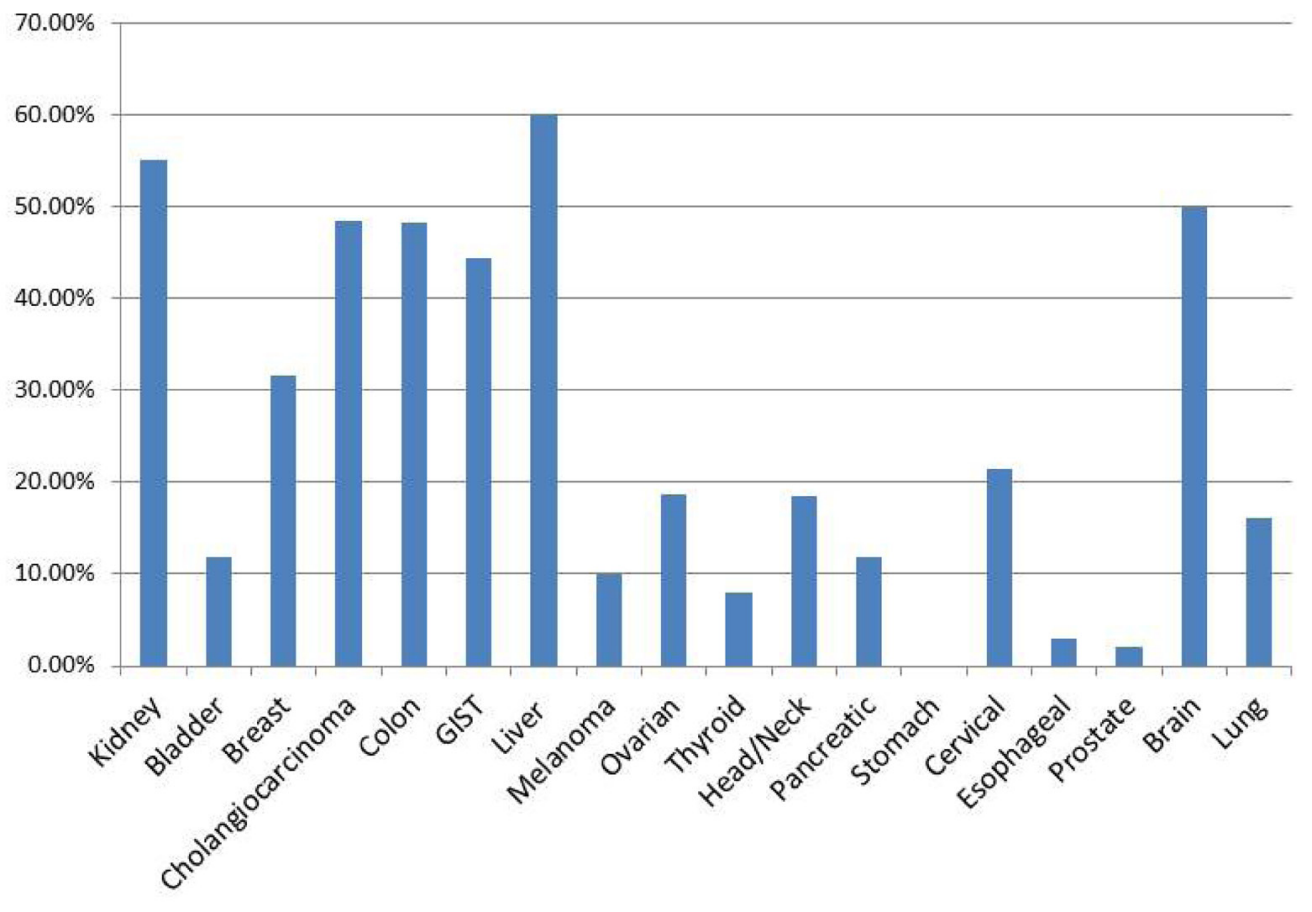
BRG1 Loss in Selected Cancer Types The 18 cancer types are shown. TMAs from each cancer type were stained with an antibody to BRG1 and scored. The y-axis represents the percentage of cases where the staining product was between 0-40.

While our analysis and those performed by Oike *et al*. [31] and Rodriquez-Nieto *et al*. [30] failed to identify mutations as a major mechanism of *BRG1* silencing, we next sought to analyze several mutation databases for the frequency of *BRG1* mutations. This allowed us to determine how *BRG1* mutation rates compare with the frequency of *BRG1* silencing by IHC. To accomplish this, we examined BRG1 expression in a variety of tumor types in order to understand the scope and breadth of *BRG1* silencing in cancer. By staining 18 different tumor microarrays (TMAs), we observed BRG1 loss greater than or equal to 10% of the tumor cells in 14 of the 18 TMAs that were analyzed (Figure 1 and Supplementary Table 2A-G), while we observed little to no negativity (i.e., no BRG1 loss) in one cancer type, stomach cancer (< 1%) (Supplementary Table 2A). We observed a ~15-40% loss of BRG1 in breast, colon, head/neck, ovarian, prostate, pancreatic, and cervical cancers (Figure 1; Supplementary Table 2A). These data demonstrate that BRG1 is lost in a broad variety of cancers. In addition, *BRG1* was observed to be silenced most predominantly in both liver (60%) and renal cell (55%) cancers. For a number of cancers that we analyzed, our TMAs were designed to include either precursor lesions or different histologic subtypes of the same tumor type. In cervical cancer, *BRG1* was observed to be silenced only in primary tumors. In contrast, in regards to the cervical cancer *in situ* samples (CIN1-CIN3), we observed that BRG1 expression was increased as a function of the degree of cervical neoplasia (Supplementary Table 2E). These data are consistent with the observation that BRG1 expression and that of its homologue BRM increase in parallel with an increasing degree of proliferation, where quiescent cells have detectible but lower levels of BRG1 expression [32]. While BRG1 loss was observed to be between 15-30% in adenocarcinoma and squamous cell carcinoma, the two predominant histologic subtypes of non-small cell lung cancer (NSCLC), we observed a low rate of BRG1 loss in other lung cancer histologies such as bronchioloalveolar carcinoma (BAC), and the neuroendocrine tumors (small cell lung cancer, large cell, carcinoid and atypical carcinoid) [33] (Supplementary Table 2B and 2C). In the brain, *BRG1* was silenced at the same frequency in Glioblastoma, Meningioma, and Astrocytoma (approximately 45-60%, Supplementary Table 2D). Supplementary Figure 1A-1D illustrates representative examples of positive, mosaic (or weak) and negative staining of tumor cores from the various TMAs that were stained.

### *BRG1* is silenced more frequently than is reported in the COSMIC and Atlas mutational databases

To further delineate if *BRG1* might be silenced by mutations, we downloaded and compiled the mutational data for various tumors and specifically examined the frequency of silent and missense mutations (which typically do not abrogate or disrupt gene expression), nonsense mutations and insertion/deletions (which typically abrogate gene expression), and total mutations for 13 tumor types from the COSMIC database and 23 tumor types from the Atlas database. We also reviewed the literature and compiled all available published IHC data and compared these data to our own findings reported in this paper. Nonsense mutations and indels, which typically result in no wild type protein expression, were compiled for bladder, breast, colon, pancreatic, prostate, melanoma and liver cancer and were found to occur in less than 1% of each tumor type. In contrast, *BRG1* is silenced in these tumors at a frequency of 12%, 32-52%, 8-48%, 12-50%, 10-67%, 10-27% and 40-60%, respectively, according to IHC. In lung adenocarcinoma, where the abrogating mutation rate was found to be the highest for *BRG1* at 3.0% (Atlas database; Table 1), the loss of BRG1 was still significantly frequent at 16-37% [30, 31, 34]. Moreover, while missense mutations can severely alter a protein's function, the rate of missense mutations in *BRG1* was < 4% and < 6% for squamous cell and adenocarcinoma subtypes of NSCLC, respectively, according to the Atlas and COSMIC databases; these values are much lower than the observed rate of BRG1 loss of expression by IHC. Similarly, for most tumors listed in Table 1, the frequency of missense mutations is approximately 2-3-fold lower than the rate of *BRG1* silencing. While *BRG1* is frequently altered or mutated in cell lines, the frequency of such *BRG1* alterations in primary tumors is too low to account for how often *BRG1* is silenced in the vast majority of these primary tumors.

### Biallelic deletions of *BRG1* do not frequently occur

**Figure 2:**
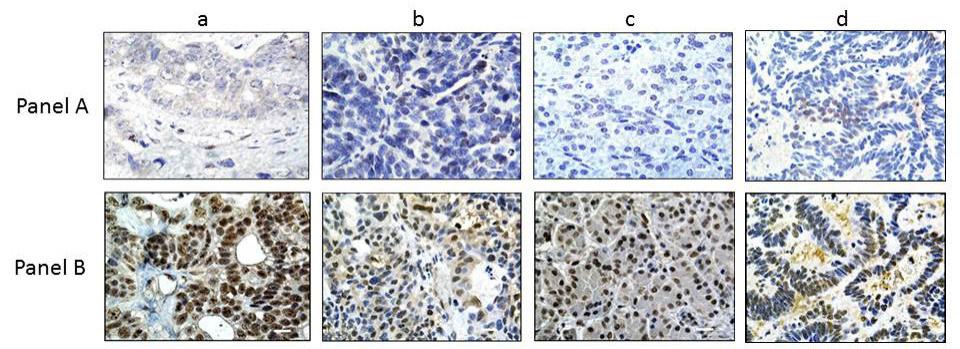
CARM1 Expression in BRG1-negative Tumors In Panel **A.**, 4 representative tumors (out of the 20 BRG-negative tumors) are shown for BRG1 IHC: a) liver tumor; b) ovarian tumor; c) pancreatic tumor; d) colon tumor. BRG1 expression by IHC was either negative or very minimal (staining product < 40) in these tumors. Panel **B.** shows the same tumors as in Panel **A.**, but stained for CARM1; as seen here, the majority of these tumors were all highly positive for CARM1 (+3, > 80%). Magnification bar = 20 μm.

We sought to determine if other mechanisms in addition to mutations might also contribute to BRG1 loss. Another mode of gene inactivation that can occur during cancer development is biallelic deletion. To this end, the *BRG1* locus has been documented to be an area of loss of heterozygosity (LOH) in a number of tumor types [25, 35] and is associated with the loss of large deletions that involve other genes in the 19p13.3 locus, such as *LKB1* [36]. As genomic deletions typically inactivate large regions that contain multiple genes [36], we reasoned that if *BRG1* was biallelically deleted, the adjacent gene *CARM1* would be frequently deleted as well. To investigate if this might occur in BRG1-deficient tumors, we performed IHC for CARM1 (*BRG1* and *CARM1* are 70 kbp apart) in each of the 20 BRG1-deficient tumors for which we had matched frozen specimens (Table 2); we observed that CARM1 was robustly expressed in > 70% of the cancer cells in each of these 20 tumors (Figure 2 Panel A (BRG1) and Panel B (CARM1)). Hence, the presence of ubiquitous CARM1 expression indicates that it is unlikely that *BRG1* is homozygously deleted in any of these 20 BRG1-deficient tumors.

**Table 2:** BRG1 is lost in a variety of tumor types, while CARM1 expression is retained

Tumor Type	BRG1-Neg. Tumors	BRG1 IHC Percent Positive (intensity)	Product	CARM1 IHC Percent Positive (intensity)
Liver (HCC)	2011	20% (1.5)	30	70% (3)
Liver (met. Colon carcinoma)	2600	0% (0)	0	90% (3)
Liver (HCC)	1825	0% (0)	0	50% (2)
Liver (HCC)	1832	15% (1)	15	50% (3)
Brain (atypical meningioma)	2459	20% (1.5)	30	80% (2)
Pancreatic	2705	20% (2)	40	80% (3)
Pancreatic	2579	30% (1)	40	85% (3)
Ovarian (endometrioid)	2359	40 (1)	40	60% (3)
Ovarian	2700	30% (1)	30	70% (3)
Omentum (met. Ovarian)	2306	20% (1)	20	70% (3)
Ovarian	2330	40% (1)	40	90% (3)
Ovarian	2472	20% (1.5)	30	80% (3)
Ovarian	2733	20% (2)	40	80% (3)
Pelvis (met. Ovarian)	2425	20% (1)	20	90% (3)
Colon Adenocarcinoma	2453	40% (1)	40	90% (3)
Colon Adenocarcinoma	2592	20% (1)	20	80% (3)
Colon Adenocarcinoma	2514	20% (2)	40	90% (3)
Colon Adenocarcinoma	2217	20% (1.5)	30	90% (3)
Colon Adenocarcinoma	2645	30% (1)	30	90% (3)
Colon Adenocarcinoma	2706	10% (1)	10	80% (3)

Tumors from the Clinical Translational Science Institute (CTSI) at the University of Florida were stained for BRG1 and 20 tumors with a staining product score = or < 40 were identified. These tumors were then analyzed for mutations and splicing defects by Sanger sequencing of the genomic DNA (BRG1 37 exons) and the mRNA, respectively. The percentage of BRG1-negative cells and the staining intensity (shown in parentheses) according to IHC are shown in the third column, while the staining product is shown in the fourth column. The same parameters are presented for CARM1 IHC where the intensity (percentage positive) and staining product are shown in the fifth and sixth columns, respectively.

Since some focal deletions in cancer can occur within small genomic areas and may result in the deletion of parts of genes rather than large chromosomal segments, we next conducted qPCR at three different points along the *BRG1* gene (*BRG1* exons 3, 18, and 36) to evaluate any deletions involving the front, end or middle of the *BRG1* gene, which spans 100 kb on chromosome 19p13.2 [35]. We compared three ΔCT values from wild type DNA (white blood cell DNA from anonymous donors; *n* = 7) with either BRG1-deficient cell lines (SW13, H522, A427, H125, H513, *n* = 5) or 20 BRG1-deficient tumors (*n* = 20). We surmised that 0, 1, and 2 allelic losses would be represented by ΔΔCT values of ~0.00, ~0.5 and > 1.0, respectively. Through a comparison of qPCR results from wild type DNA with cell line DNA, we found no allelic loss for H513 (*p* = 0.51 ΔΔCT = 0.07). In contrast, we found that SW13 harbors a single *BRG1* allelic loss (*p* < 0.02, ΔΔCT = 0.50) and that A427 harbors biallelic loss in the proximal part of the *BRG1* gene (*p* < 0.01, ΔΔCT = 3.29), which is consistent with previous work by Wong *et al.* [20]. Similarly, after a comparison of qPCR values for BRG1-deficient tumors with wild type control DNA, only one tumor showed a statistically significant difference (*p* = 0.001), where the ΔΔCT was equal to 1.85, which is consistent with focal biallelic loss of *BRG1*. The other 19 BRG1-deficient tumors had ΔΔCT < 0.09 (*p* values > 0.5-0.7) with no detectible *BRG1* allelic loss. These data show that biallelic deletion can occur infrequently in BRG1-deficient tumors (~5%).

### Aberrant splicing silences *BRG1*

**Figure 3:**
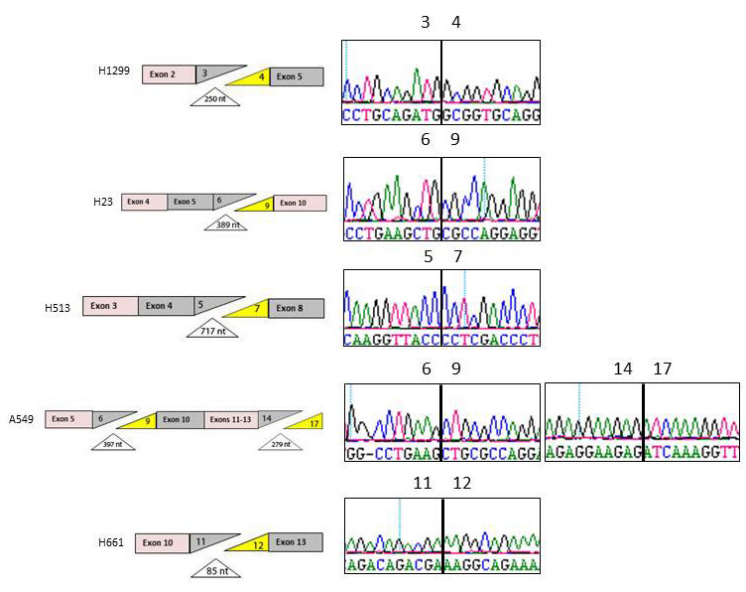
Splicing Defects in BRG1-negative Cell Lines PCR amplification of the mRNA using 5 amplicons spanning the BRG1 mRNA revealed bands that were either shorter or longer than the wild type bands. Sequencing of these bands revealed the omission of certain exons and/or the addition of intron sequences resulting in aberrant splicing. The diagram on the left illustrates which exons were omitted from 5 BRG1-deficient cell lines and the diagram on the right shows the chromatograph of the resultant hybrid mRNA.

In order to determine the primary mechanism of *BRG1* silencing in tumors, we analyzed and sequenced the mRNA from 16 BRG1-deficient cell lines for BRG1 using five nested PCR overlapping amplicons (Supplementary Table 1B). Our analysis of BRG1-deficient cell lines has revealed mutations as a major underlying mechanism of *BRG1* silencing in these cells, since 7 out of 16 (~44%) cell lines harbored nonsense or insertion/deletion (indels) mutations (Table 3). However, 6 out of 7 mutations have been previously reported [20, 21]. In addition to these alterations, our sequencing of *BRG1* from these cell lines revealed a series of as yet unreported splicing defects in 7 of these BRG1-deficient cell lines (~44%). The splicing defects were most common between exons 3 and 8, as illustrated in Figure 3 and Supplementary Figure 2. In each of these cases, the splicing defects caused a frameshift upstream of the helicase domain, which assures the disruption of BRG1 function. Hence, these data revealed that splicing defects in BRG1 may be a potential mechanism that underlies *BRG1* silencing in primary tumors. To determine what might cause the observed aberrant splicing, we sequenced each of the *BRG1* exons using genomic DNA in order to examine the splicing acceptor and donor sites as well as the branch-chain site. None of these sites in any of the 7 cell lines showed any alterations or mutations in these splicing associated-DNA segments (data not shown). In addition to the cell lines with either mutations (i.e., indels or nonsense) or splicing defects, 3 BRG1-deficient cell lines (C33A, Panc-1, and H1573) out of 17 were devoid of any identifiable alterations that could account for why BRG1 expression was absent by Western blot (Figure 6; Table 3).

**Table 3:** Deletions, mutations and splicing defects in BRG1-negative cell lines

Cell Line	Tumor Type	Defect in BRG1	Location	Reporting Author
UMSCC-6	Head/Neck	Splicing defect	Missing Exon 6	
UMSCC-14A	Head/Neck	Splicing defect	Missing Exon 5-8	
UMSCC-22B	Head/Neck	Splicing defect	Missing Exon 6/7	
H23	Lung	Splicing defect	Missing Exon 7-10	
A427	Lung	Truncated 3100-4842	Frameshift	Wong, Medina
H661	Lung	3476delG & splicing defect	Frameshift; Missing part of exon 11	Medina
H1299	Lung	Splicing defect & Truncation 69bp	Parts of exon 3/4	Wong, Medina
A549	Lung	Splicing defect	Missing Exon 7/8; 15/16	Medina
H125	Lung	E1056X; nonsense mutation	Frameshift	
H513	Lung	Splicing defect	exons 6-8	
H522	Lung	805_806Del	Frameshift	Medina
H157	Lung	169DelC	Frameshift	Medina
SW13	Adrenal	Nonsense Q164X	Truncated	
Panc-1	Pancreatic	No Alteration detected	Epigenetic	
H1573	Lung	No Alteration detected	Epigenetic	
C33A	Cervical	No Alteration detected	Epigenetic	Wong

The mRNA from 16 BRG1-deficient cell lines was sequenced using overlapping nested PCR amplicons. Mutations and splicing defects were identified in 13 of these cell lines as listed in the table, while no detectible alterations were detected in 3 cell lines: C33A, Panc-1 and H1573.

**Figure 4:**
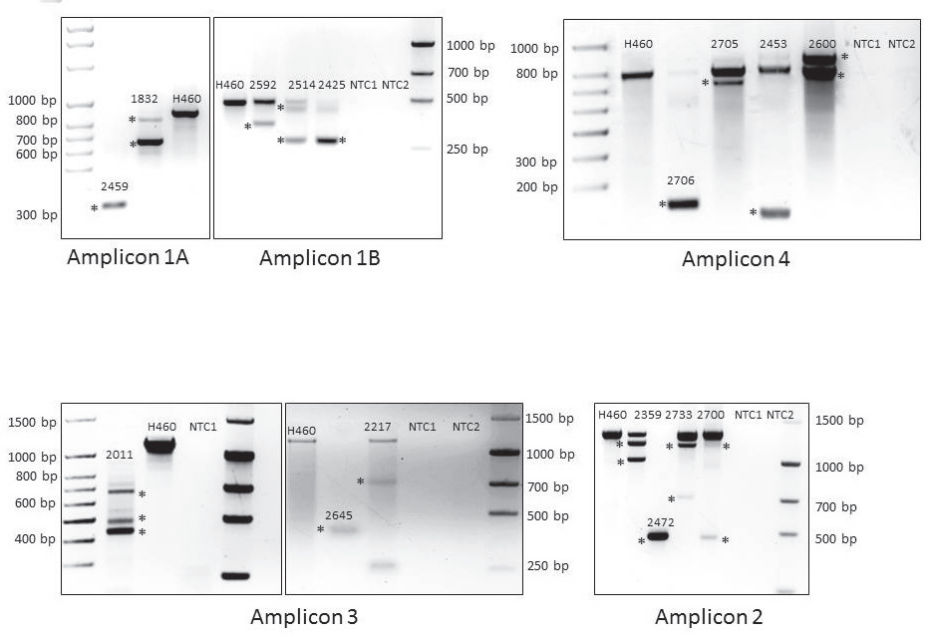
BRG1-Negative Primary Tumors Show Aberrant BRG1 Splicing Amplification of the 1A, 1B, 2, 3 and 4 amplicons are shown in a positive control cell line (H460: wild type band) along with those specific BRG1-deficient primary tumors that show altered splicing. The chromatographs for each aberrantly spliced BRG1 transcript are shown in Supplementary Figure 3.

Based on our findings of splicing defects in these BRG1-deficient cell lines, we next sought to analyze primary BRG1-deficient tumors for the presence of aberrant BRG1 splicing. To accomplish this, we identified 20 tumors (paraffin-embedded sections) that each had little to no BRG1 expression by IHC and where we also had matched frozen tumor specimens. By hematoxylin and eosin (H&E) staining (data not shown), each of the matched frozen tumors was found to contain greater than 90% tumor, and after staining for BRG1, these specific samples were also found to be BRG1-deficient, which is consistent with IHC of their paraffin-embedded counterparts. We then isolated mRNA from all 20 matched BRG1-deficient frozen tumors and then amplified the BRG1 mRNA using 5 nested overlapping PCR amplicons (Supplementary Table 1B) to determine if any aberrant splicing defects could be found similar to what was observed in BRG1-deficient cell lines. We found that 16 tumors (80%) harbored some degree of aberrant splicing (Supplementary Figure 3) whereas complete aberrant splicing (without any wild type band observed) was found in only 8 tumors (40%) (Figure 4; Table 4). An examination of the genomic sequence data of the exons that flank each aberrant splicing junction revealed no mutations in the acceptor, donor, or branch sites.

**Table 4:** BRG1-negative tumors have splicing defects

Tumor	BP	AA	AA	Amino Acids	Exons	Domains	In/out	Inactivate	Epitope	Wild type
	Deleted	Start	End	Deleted	Effected	Deleted	Frame	Protein	Present; IHC	Band
1832	237	78	157	79	4-5	--	In	No?	No	No
2459	616	29	234	205	3-5	QLQ	Out	yes	No	No
2011	909	853	1156	303	19-26	Dead box; helicase	In	yes	Yes	No
2217	861	851	1138	287	19-26	Dead box; helicase	In	yes	Yes	Yes
2306	861	851	1138	287	19-26	Dead box; helicase	In	yes	Yes	Yes
2359	273	654	745	91	16-17	--	In	No?	Yes	yes
2514	127	360	402	42	8	--	Out	yes	Yes	No
2425	127	360	402	42	2	--	Out	yes	Yes	No
2453	783	1134	1395	261	27-32	SnAC	Out	yes	Yes	Yes
2705	96	1391	1423	32	31	--	Out	yes	Yes	Yes
2706	753	1219	1470	251	27-32	SnAC	In	yes	Yes	No
2600	100	774	807	33	28-29	--	Out	yes	Yes	Yes
2645	712	876	1113	237	20-25	Dead box	Out	yes	Yes	No
2700	831	530	807	277	11-17	DEADc; BRK	In	yes	Yes	Yes
2472	818	543	816	273	11-17	DEADc; BRK	Out	yes	Yes	No
2733	123	667	708	41	15	--	In	No?	Yes	Yes

Each primary tumor found to harbor splice variants is presented. The number of base pairs (bp) deleted is shown in the second column. In the third and fourth columns, the amino acid (AA) start and end values are given, respectively, followed by the number of amino acids that are deleted in the fifth column. The exons that are spliced out partially or completely in a given tumor are indicated in the sixth column whereas the domains of the BRG1 protein that are affected by the splicing defects are listed in the seventh column. Whether or not the splicing defects resulted in the frame-shift of the protein and the inactivation of the protein are indicated in the eighth column. The ninth column lists whether or not BRG1 would be expected to be inactivated due to this aberrant splicing event. In addition, the tenth column lists whether or not the BRG1 antibody epitope might be expected to be omitted by the aberrant BRG1 splicing. Presence of a wild type band observed after nested PCR of the cDNA is shown in the eleventh (last) column. The shading indicates the tumors in which loss of BRG1 expression would be expected to occur.

To establish which of these splicing defects might result in the loss of BRG1 function, we examined the location of the splicing defects to determine if this observed aberrant splicing might result in the loss of any critical BRG1 domains. Table 4 shows that in 13 out of 16 tumors, the resultant defective splicing causes a frameshift in the BRG1 transcript upstream of one or more functional critical domains such as the SnAC, or Deadbox/helicase domain, which would cause a loss of BRG1 function [37] [38, 39]. Aberrant BRG1 splicing occurred in-frame and did not disrupt a required functional domain in only 3 tumors. Therefore, aberrant BRG1 splicing is an unreported mechanism that may cause *BRG1* silencing in a subset of BRG1-deficient tumors.

### BRG1 loss is associated with E-cadherin loss and an increase in Vimentin expression

**Figure 5:**
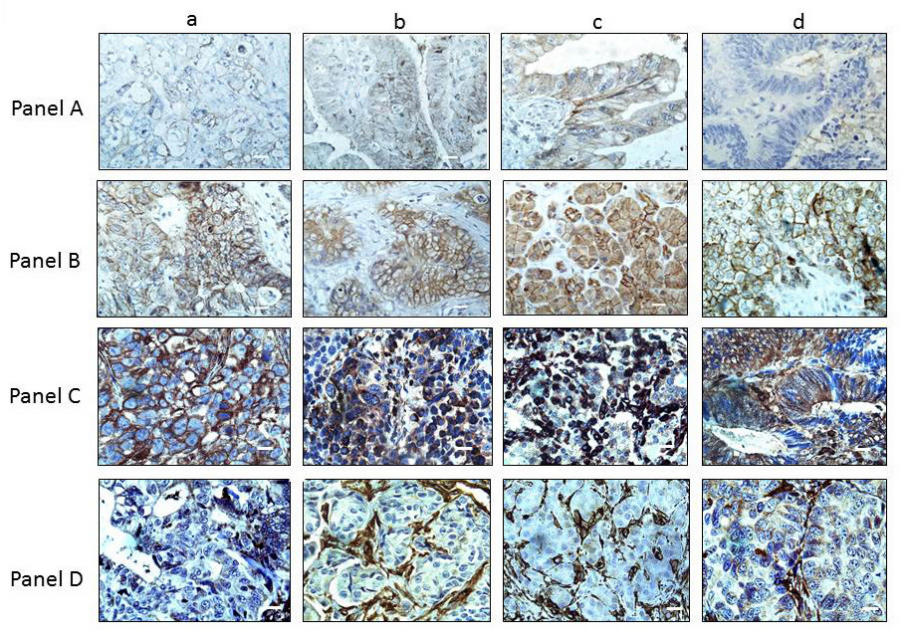
BRG1 Loss Drives E-cadherin Downregulation and Vimentin Upregulation Panels A and **B.** show four BRG1-negative and BRG1-postive tumors, respectively, stained for E-cadherin (a) liver, (b) ovarian, (c) pancreatic and (d) colon tumors. Panels **C.** and **D.** show the same four BRG1-negative and four BRG1-positive tumors, respectively, stained for vimentin. Note that in Panel **D.**, vimentin is expressed in normal cells such as fibroblasts and in the tumor stroma. Magnification (white bars) = 20 μm.

BRG1 loss can potentially impact cancer development, as a variety of key cellular proteins are SWI/SNF-dependent, such as the transcription factor ZEB1, which regulates E-cadherin and epithelial to mesenchymal transition (EMT) [40, 41]. Similarly, BRG1 loss has been tied to another protein, Vimentin, whose up-regulation is linked to EMT and metastatic phenotypes. To this end, SWI/SNF has been shown to regulate E-cadherin and Vimentin expression in cell lines [40–42]. As E-cadherin loss and the up-regulation of Vimentin are strongly associated with metastatic behavior and worse patient survival [43–45], the loss of BRG1 might affect cancer development by facilitating the loss of E-cadherin and the up-regulation of Vimentin in primary tumors as well as those with a metastatic phenotype. We stained the 20 BRG1-negative and 20 (matched for tumor type) BRG1-positive tumors for E-cadherin and Vimentin expression and found that BRG1 loss was correlated with a loss of E-Cadherin expression and an increase in Vimentin expression by IHC (Figure 5). We then performed qPCR on the total RNA from the 20 BRG1-negative and 20 BRG1-positive tumor specimens and found that BRG1 loss was statistically associated with E-cadherin loss (4-fold difference) in these primary tumors (two-tailed *t*-test, *p* = 1.9E-03). Similarly, BRG1 loss was also statistically correlated with increased expression of Vimentin (~4-fold; two-tailed *t*-test, *p* = 8.4E-04), and the largest changes were observed in liver and colon tumors (10-fold and 6-fold, respectively; *p* = 4E-03 and *p* = 2E-02, respectively). Hence, in both cell lines and primary tumors, BRG1 loss is correlated with the up-regulation of Vimentin and the loss of E-cadherin. This is important because these data help to explain why BRG1 loss in primary tumors is associated with a poor prognosis, as seen in primary breast cancers and other cancer types [34, 46, 47]. Moreover, the changes in both E-cadherin and Vimentin expression not only support the loss or decrease of BRG1 expression, but they also indicate a loss of BRG1 function in the majority of these 20 BRG1-deficient tumors.

### AKT activation drives *BRG1* silencing in cancer cell lines

**Figure 6:**
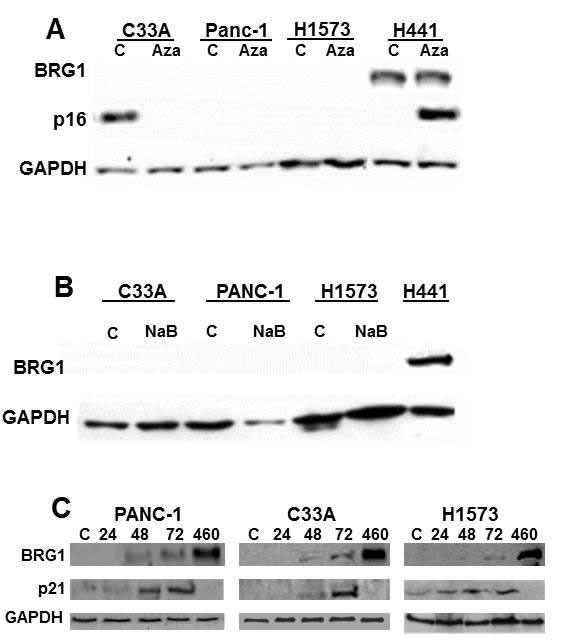
BRG1 is not Induced by Inhibitors of Epigenetic Mechanisms **A.** BRG1-deficient cell lines C33A, Panc-1, and H1573 were treated with 5-Aza-deoxycytidine at 5 μM (a cytidine analogue that inhibits DNA methylation) daily for 3 days. In H441 cells, the induction of the methylated p16 served as a control for the 5-azadeoxycytidine treatment. **B.** These 3 cell lines were also treated with 5 μM sodium butyrate (NaB), an HDAC inhibitor, for 3 days. **C.** These cell lines were treated with 10 μM of the proteasome inhibitor MG-132, and a slight induction of BRG1 was observed at both 48 and 72 hours after treatment. p21, a known BRG1-dependent gene, was observed to be induced along with BRG1.

To determine how *BRG1* might be silenced by a means other than mutations and splicing defects, we examined 3 BRG1-deficient cell lines [10, 48] that lack any definite alterations such as splicing defects or mutations (C33A, Panc-1, H1573). We then specifically examined whether *BRG1* might be reversibly or epigenetically silenced in these 3 cell lines by testing a variety of compounds known to reverse epigenetic silencing [49–51]. Specifically, we treated each of these cell lines with the following compounds: 5-Aza-deoxycytidine (Decitabine) to reverse DNA methylation, Sodium Butyrate (NaB; a pan HDAC inhibitor) to maintain protein acetylation (histone acetylation is known to control gene expression) or the proteasome inhibitor MG-132 to prevent protein degradation. The treatment with Decitabine at 5 μM for 72 hours did not exert any effect on BRG1 expression in any of these 3 nonmutated BRG1-deficient cell lines (Figure 6A). As a control, parallel treatment of the lung cancer cell line H441 with Decitabine resulted in the induction of p16 protein, which is silenced by DNA methylation in this cell line. Similarly, 5 μM NaB for 72 hours also exerted minimal to no impact on BRG1 expression in each of these 3 cell lines (Figure 6B) but was sufficient to induce BRM (the homologue of BRG1) in these cell lines [52]. In contrast, treatment with the proteasome inhibitor MG-132 at 10 μM for 24, 48 and 72 hours slightly induced BRG1 protein expression compared with the positive control cell line H460 and the untreated BRG1-deficient cell lines (Figure 6C). Upon treatment with MG-132, we also observed a parallel induction of p21, which is a BRG1-dependent gene (Figure 6C) [53]. Therefore, this slight induction suggests that *BRG1* is probably reversibly silenced and that the BRG1 protein is functional in these 3 cell lines.

**Figure 7:**
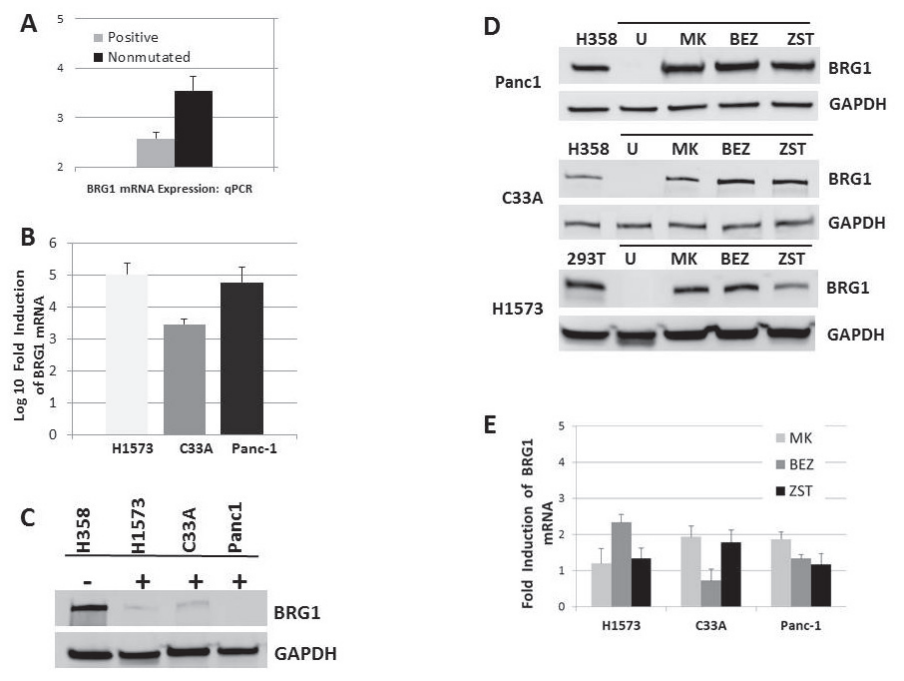
BRG1 is Silenced by the PI3K/AKT/mTOR Pathway at the Translational Level **A.** shows that the BRG1 mRNA levels only differ by about ~1.2 ΔCT value (equal to around a ~2.3-fold difference) in BRG1-positive and BRG1-negative (nonmutated) cell lines. **B.** BRG1 transfection into C33A, Panc1 and H1573 cells caused greater than 1000-fold induction of BRG1 mRNA relative to untransfected control. **C.** After BRG1 transfection into C33A, Panc1 and H1573 cell lines, BRG1 protein only marginally increased BRG1 untransfected is denoted by “-“ and BRG1 transfected cells are denoted by “+”. **D.** BRG1 induction was observed when Panc1, C33A and H1573 cells were treated with 5 uM of the pan AKT inhibitor MK-2206 (MK), the dual PI3K/mTOR inhibitor BEZ235 (BEZ) or the PI3K inhibitor ZSTK474 (ZST) for 72 hours. “U” designates the test cell line: C33A, Panc1 or H1573 without drug treatment. GAPDH was used as the loading control. **E.** BRG1 mRNA only increased ~2-fold after treatment with MK2206, BEZ235 or ZST474 in the C33A, Panc1 and H1573 BRG1-deficient cell lines.

In order to better determine how *BRG1* might be regulated, we compared the level of BRG1 mRNA by qPCR in BRG1-positive (*n* = 14), and BRG1-negative (nonmutated: *n* = 13) cell lines. We observed in BRG1-positive and BRG1-negative (nonmutated) cell lines that BRG1 mRNA differed by ~1.2 ΔCT, which represents an approximately 2.3-fold difference (Figure 7A), although the levels of BRG1 protein in these two groups typically differed by > 10-20 fold (Figure 6A-6C). In addition, if BRG1-negative nonmutated cell lines are transfected with BRG1, the mRNA is observed to increase > 1000 fold, while the BRG1 protein levels are marginally detectible by western blot (Figure 7B and 7C). These data illustrate a discordance between BRG1 mRNA and BRG1 protein levels suggesting that BRG1 is regulated at the translational level. We next tested a kinase inhibitor library containing 140 different inhibitors (from Cayman Chemicals, Ann Arbor, MI, USA) to determine if the abrogation of some of the commonly studied kinases might be able to induce BRG1. We found that a pan AKT pathway inhibitor (MK2206), a PI3K inhibitor (ZSTK474) and a dual PI3K/mTOR inhibitor () could all readily induce BRG1 in these 3 BRG1-deficient cell lines (Figure 7D). Interestingly, the BRG1 mRNA levels in these cell lines only slightly increased (~2 fold) (Figure 7E) after the application of these inhibitors, while in contrast, the BRG1 protein levels were significantly elevated by these PI3K/AKT/mTOR pathway inhibitors. These data (Figure 7D and 7E) again suggest the assertion that BRG1 is regulated at the translational level. These data suggest that BRG1 is regulated at the translational level. *BRG1* is reversibly silenced in a subset of cancer cell lines, and compounds that are typically used to reverse epigenetic silencing had little to no effect on BRG1 expression. On the contrary, inhibitors of the PI3K/AKT/mTOR pathway were observed to be effective in the restoration of BRG1 protein expression in these cell lines.

## DISCUSSION

While cell line analyses of *BRG1* alterations clearly demonstrate the presence of abrogating mutations in the majority of BRG1-deficient cell lines, such data in primary tumors are largely absent. The strongest data show that mutations are not the major mechanism of *BRG1* suppression, as is seen in the comparison of BRG1 loss by IHC with the mutation data from the COSMIC and Atlas databases [28]. Similarly, while NGS studies have revealed that mutations do occur in almost all SWI/SNF subunits, including *BRG1*, the rate of total mutations as well as the rate of abrogating mutations in *BRG1* are far below the rates of documented loss of BRG1 in primary tumors as observed by IHC [28]. One reason for the wide range of loss in certain tumor types is due to the IHC scoring methods used by different investigators; we consider a staining product of 0-40 as negative, whereas others may use a different scoring system or may consider “weak” expression as “negative”. Moreover, abrogating splicing defects have been documented to occur in cell lines as a mode of *BRG1* silencing. It is important to note that the short reading methods employed by most NGS protocols cannot efficiently detect the occurrence of aberrant splicing [54–56]. Thus, this helps to explain why BRG1 splicing defects have not yet been frequently reported in the literature. BRG1 splicing defects were also documented in our analysis in a subset of tumors, which indicates that this mode of *BRG1* silencing occurs, but based on our representative sample, it does not occur frequently enough to account for how often *BRG1* is silenced in the vast majority of BRG1-deficient tumors.

Normal tissue and islands of BRG1-positive tumor cells (and BRG1-positive internal control cells) can be present in the tumor samples. If the mRNA from normal tissue is more prevalent than the tumor mRNA, it may be more difficult to detect aberrant BRG1 splicing in certain cases. The frequency of detection of aberrantly spliced BRG1 mRNA is likely an underestimation, particularly in those tumors where the BRG1 pattern of loss/expression resembled a more mosaic pattern. In contrast, NGS of tumor DNA, which is proportional to the amount of tumor present rather than the level of total mRNA, has shown mutations at the ends of *BRG1* exons (splicing donor sites). Mutations in these areas might impact BRG1 splicing and therefore support this mode of *BRG1* suppression. Since we found a lack of definite mutations and found splicing defects only in a subset of primary tumors, this prompted us to investigate whether *BRG1* might be epigenetically silenced by activation of the AKT pathway. However, unlike the epigenetic silencing of *BRM*, the homologue of *BRG1* (i.e., the other SWI/SNF ATPase or catalytic subunit), where *HDAC9* is concomitantly over-expressed [57], we have yet to detect a marker to delineate the occurrence of the epigenetic silencing of *BRG1*.

Various investigators have examined different tumor types for alterations in *BRG1* using an array of different experimental methods. For example, Medina *et al*. [58] examined 70 primary lung tumors and found a complete absence of any abrogating mutations by Sanger sequencing. Given that *BRG1* is silenced in ~16% of primary lung cancers [7], the random sequencing of 70 lung cancers conducted by Medina *et al*. [58] would have been expected to identify 7-11 tumors with mutations (even if the tumors had been chosen at random). However, silencing alterations (e.g., nonsense mutations and indels) were not found. Valdman *et al*. performed a complete mutational analysis of all 37 *BRG1* exons in 21 prostate tumors and found a complete lack of mutations [24], although BRG1 loss ranges from ~15% to 50% by IHC [59–61]. Sentani *et al.* looked for mutations in the *BRG1* gene in 8 gastric carcinoma cell lines and 33 primary gastric carcinomas by PCR-single-strand conformation polymorphism (SSCP) analysis, and no SSCP variants were found [62]. Endo *et al*. observed copy number losses of the *BRG1* and *BRM* genes in 14 (26%) and 7 (13%) of 54 primary HCC tumors, respectively. They only found 4 somatic missense mutations in the *BRG1* gene in 2 of 36 primary HCC tumors but found no abrogating mutations that could explain the loss of BRG1 in HCC even though BRG1 expression is absent in ~60% of HCC tumors according to IHC. Similarly, Oike *et al.* analyzed 101 cases of NSCLC, where 16 (13%) were found to be BRG1-deficient; additionally, the sequencing of the genomic DNA from the 16 tumors for *BRG1* revealed that none of these tumors harbored any abrogating mutations [31]. Rodriguez-Nieto *et al.* stained 122 tumors and found that 46 (37%) were either low or negative for BRG1 [30]. Twelve of these tumors were analyzed for *BRG1* mutations by Sanger sequencing and only a single nonsense mutation was found. The combination of the available published data in NSCLC for *BRG1* mutations by Oike *et al*. and Rodriguez-Nieto *et al*. reveals that 1 of 28 (3.5%) NSCLC tumors had abrogating mutations in *BRG1* [30, 31]. These findings are consistent with mutational data from the Atlas and COSMIC databases where the total rates of abrogating mutations in *BRG1* are 3.7% and 4.2%, respectively [28].

Available IHC data show how frequently *BRG1* is silenced in primary tumors. However, the methodologies of how IHC scoring is performed and the reported end points introduce some variables in the frequency of BRG1 loss. In many cases, BRG1 expression is mosaic, where tumor cells with a complete absence of BRG1 expression are juxtaposed and intermixed with tumor cells that are robustly positive for BRG1. Therefore, while BRG1 expression may be nearly absent in a subset of tumor cells, it is difficult to determine if a particular tumor is deficient or positive for BRG1 since subsets of both positive and negative tumor cells may be found in a given tumor. Moreover, many tumors show a trace low level of BRG1 expression in a majority of tumor cells within a given tumor. This appearance of BRG1 expression is consistent with an epigenetic mode of *BRG1* regulation. In contrast, we found that BRG1 splicing is most often observed in those tumor specimens that are relatively devoid of BRG1 expression in any tumor cells. Moreover, the presence of islands and pockets of BRG1-positive cells amidst a relatively BRG1-deficient tumor background indicates an evolving tumor cell population where some cells express BRG1 and some do not. These realities attest to the heterogeneity within tumors and the difficulties that invariably occur when a single experimental approach is applied.

*BRM* is known to be acetylated when its growth inhibitor properties are turned off [63]. Similarly to BRM, the anticancer properties of BRG1 are also likely to be inactivated by some type of post-translational modification, although we do not yet completely understand how this might occur. We do know that both BRG1 and BRM can be phosphorylated prior to entry into mitosis, which causes BRG1 and BRM to translocate from the nucleus into the cytoplasm [64]. Moreover, like BRM, which is regulated by the MAPK pathway [57], BRG1 appears to be controlled by a highly related and tangential pathway, the AKT pathway. However, unlike BRM, where we have established that blocking the MAPK pathway can both induce BRM as well as cause its de-acetylation [57], which both lead to the activation of BRM, we have only found thus far that BRG1 can be induced when the AKT pathway is inhibited. If the parallels between BRG1 and BRM hold, however, we surmise that inhibition of the AKT pathway would also change the post-translational modification of BRG1 and thereby also activate its growth-inhibiting properties. Further investigations will clarify the major mechanism that drives the loss of BRG1 expression in tumors, if and how BRG1 is post-translationally modified, and if these changes also subvert the anticancer properties of BRG1.

## MATERIALS AND METHODS

### Tissue samples and immunohistochemistry

Paraffin-embedded sections of human tumors were obtained from the Clinical and Translational Science Institute (CTSI) at the University of Florida. Sections were stained according to established protocols and as described in our previous publications [32, 65]. For BRG1 IHC, a mouse monoclonal antibody (a gift from Pierre Chambon) was used for the initial IHC experiments since it was previously determined to be specific for BRG1. In order to confirm the specificity of the staining, we also used the mouse monoclonal antibody sc-374197 at a 1:100 dilution (Santa Cruz Biotechnology, Dallas, TX, USA) and the rabbit polyclonal antibody 21634-1-AP at a dilution of 1:200 (21634-1-AP, Protein Tech, Chicago, IL, USA). Anti-mouse and anti-rabbit biotinylated secondary antibodies were used at a dilution of 1:200, followed by incubation with HRP-Streptavidin at a concentration of 1:200 (SA-5004, Vector Labs, Burlingame, CA, USA). DAB (3,3′-Diaminobenzidine) was used as the chromogen (cat. # 550880, BD Pharmingen, San Jose, CA, USA), and Harris hematoxylin was used as the counterstain. All matched frozen tissues were sectioned on a cryostat and stained with hematoxylin and eosin to ensure that the sample contained > 80% tumor tissue. IHC with an antibody to CARM1 (ab110024, Abcam, Cambridge, MA, USA) was also performed in this study. To score the BRG1-negative tumors, we used a standard scoring system where the intensity of staining was scored 0-3 and the percentage of positive cells was scored 0-100%. Based on the product of the intensity and the percentage of positive cells, each tumor was assigned a product score where 0-50 indicates no expression, 50-100 indicates low expression, 100-200 indicates moderate expression, and 200-300 indicates high expression. The lung cancer TMAs were generated by Dr. Reisman's laboratory, but all other TMAs were a gift from Dr. Thomas Giordano at the University of Michigan Department of Pathology. The number of tumors stained for each tumor type is listed Supplementary Data 2A-2D.

### Western blot

Western blot analysis was conducted as previously described [66, 67]. Briefly, whole-cell lysates were extracted in urea lysis buffer (8.8urea, 5NaH_2_PO_4_, 1Tris, pH 8.0) and stored at −80°C for future use. A total of 80 μg of protein was mixed with 6x Lamelli buffer and boiled for 10 minutes. Protein extracts were run on a 4-15% pre-cast polyacrylamide gel (Bio-Rad, Hercules, CA, USA) for 1 hour at a constant voltage of 150 V. Proteins were then transferred onto an Immobilon-P membrane (Millipore, Billerica, MA, USA) for 1 hour at a constant current of 350 mA. The primary antibodies were as follows: monoclonal anti-BRG1 antibody (sc-17796, 1:200, Santa Cruz Biotechnology); monoclonal mouse anti-p21 (556430, 1:500, BD Pharmingen, San Jose, CA, USA); polyclonal rabbit anti-p16 (10883-1-AP, 1:500, Protein Tech, Chicago, IL, USA). Glyceraldehyde 3-phosphate dehydrogenase antibody (GeneTex Inc., Irvine, CA, USA) was used as the loading control. Anti-mouse and anti-rabbit secondary antibodies were purchased from GE Healthcare (Buckinghamshire, England, UK). Western blots were developed using an ECL Prime Western blot detection kit (GE Healthcare) and were analyzed with ImageJ software (National Institutes of Health (NIH), Bethesda, MD, USA).

### RNA and DNA purification, PCR and sequencing

For purification of the genomic DNA, frozen tumor samples were sectioned and incubated in ATL buffer and Proteinase K (Qiagen) overnight at 56°C. A Qiagen DNeasy Blood and Tissue kit was then used according to the manufacturer's instructions (Qiagen, Valencia, CA, USA). For the purification of RNA, frozen samples were sectioned and placed in TRIzol reagent (Life Technologies, Grand Island, NY, USA), and the total mRNA was isolated using the Sigma RNA extraction kit (Sigma Aldrich, St. Louis, MO, USA) according to the manufacturer's instructions. Reverse transcription was performed with the Superscript III First Strand Synthesis kit (Invitrogen, Carlsbad, CA, USA). The primer sets that were used to amplify the 37 exons of BRG1 are shown in Supplementary Table 1A. The primers that were used for the nested PCR are listed in Supplementary Table 1B. For the PCR of genomic DNA, the following reaction conditions were used: 95°C for 2 minutes followed by 34 cycles at 95°C for 30 seconds each, 59.5°C for 40 seconds, 72°C for 1 minute and 72°C for 7 minutes. For nested PCR of the cDNA, the following reaction conditions were used: 95°C for 2 minutes followed by 35 cycles at 95°C for 30 seconds each, 63°C for 40 seconds, 72°C for 40 seconds and 72°C for 5 minutes. All Sanger sequencing reactions of the cDNA and the amplified genomic DNA were performed by Genewiz (Genewiz, Boston, MA, USA).

## SUPPLEMENTARY MATERIALS FIGURES AND TABLES



## Abbreviations

BRG1: Brahma-related gene 1
IHC: Immunohistochemistry
PCR: Polymerase chain reaction
TCGA: The Cancer Genome Atlas
COSMIC: Catalogue of Somatic Mutations in Cancer
EMT: Epithelial to mesenchymal transition
TMA: Tumor microarray
NGS: Next Generation Sequencing
