# Supplementary material for: Mechanism of BRG1 silencing in primary cancers
Source: Oncotarget. 2016 Jul 13;7(35):56153–69. doi: 10.18632/oncotarget.10593 (PMC5302903; doi:10.18632/oncotarget.10593)
Supplement: Supplementary file 1 [file oncotarget-07-56153-s001.pdf]

Mechanism of BRG1 silencing in primary cancers

Supplementary Material

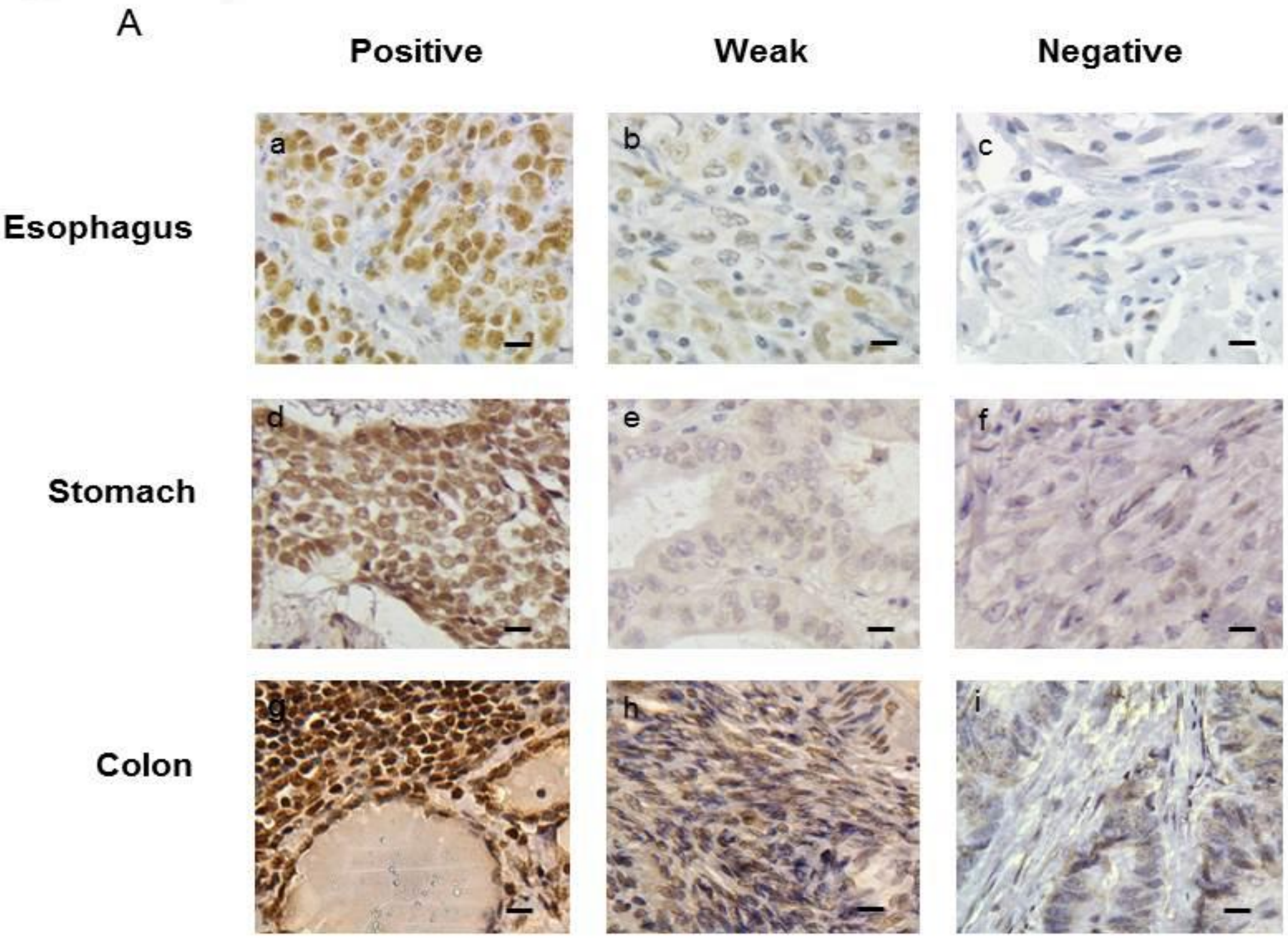

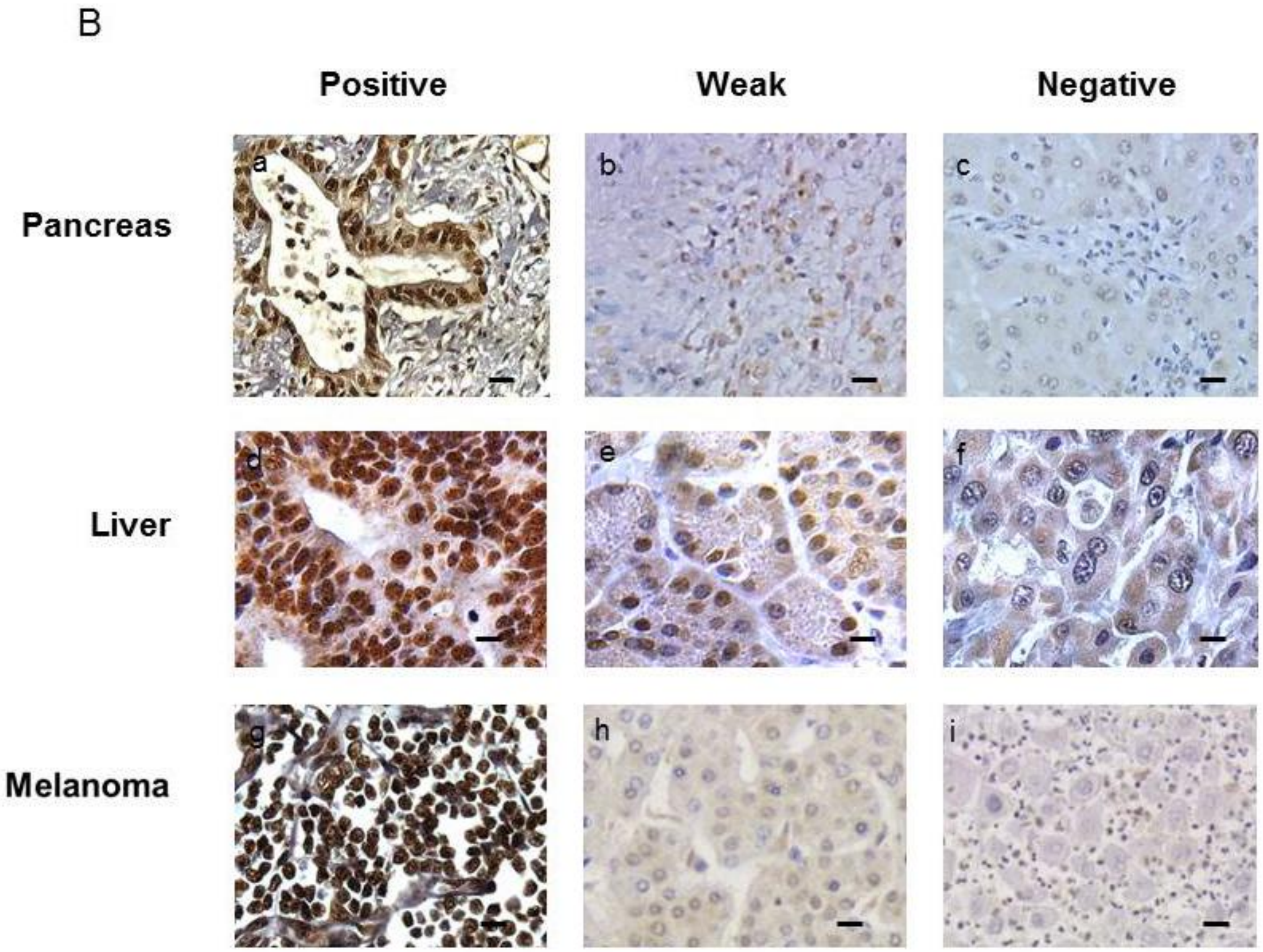
B
Positive
Weak
Negative
Pancreas
Liver
Melanoma
a
b
c
d
e
f
g
h
i

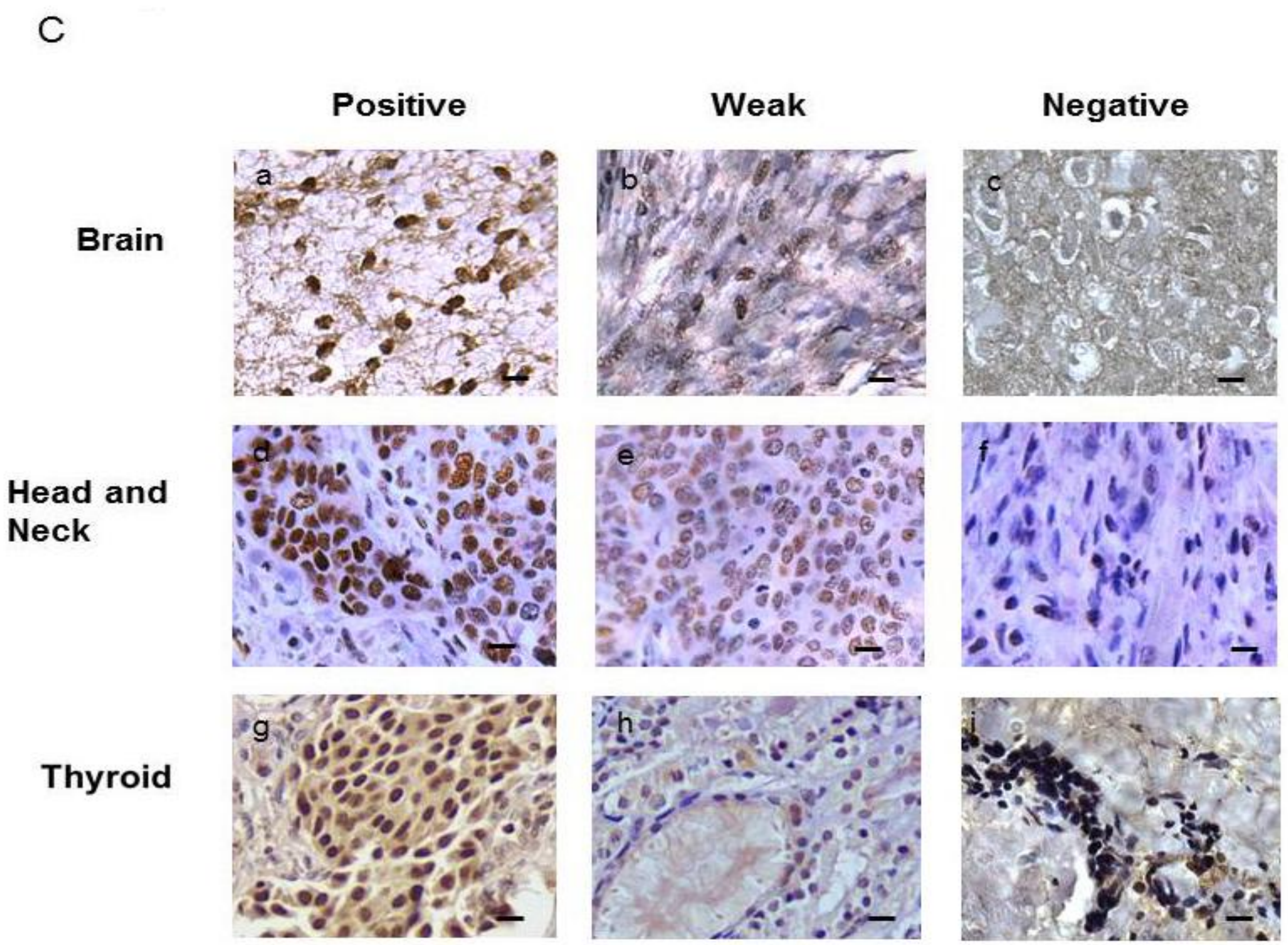
C
Positive
Weak
Negative
Brain
Head and Neck
Thyroid
a
b
c
d
e
f
g
h
i

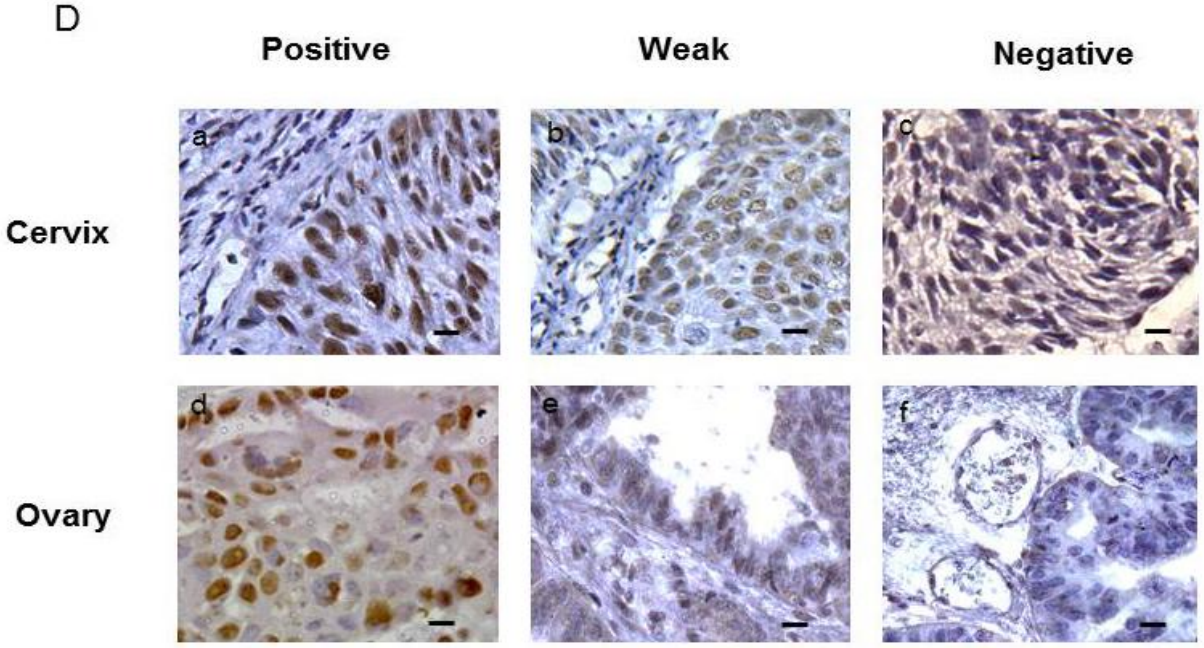

Supplementary Figure 1A-D ***Patterns of BRG1 loss in human tumors.*** TMAs of various human tumor types were stained by IHC and scored as previously described. Panels A-D feature representative images of tumors that were considered strong (staining product 201-300), weak-moderate (staining product 41-100 and 101-200) and negative (staining product 0-40) for BRG1 by IHC. Panel A: esophageal, gastric and colorectal tumors that are positive, weak and negative for BRG1. Panel B: pancreatic cancer, liver cancer and melanoma. Panels C and D: brain, head/neck and thyroid cancers and cervical and ovarian cancers. All images were obtained at 63x magnification. Bar = 20 µm.

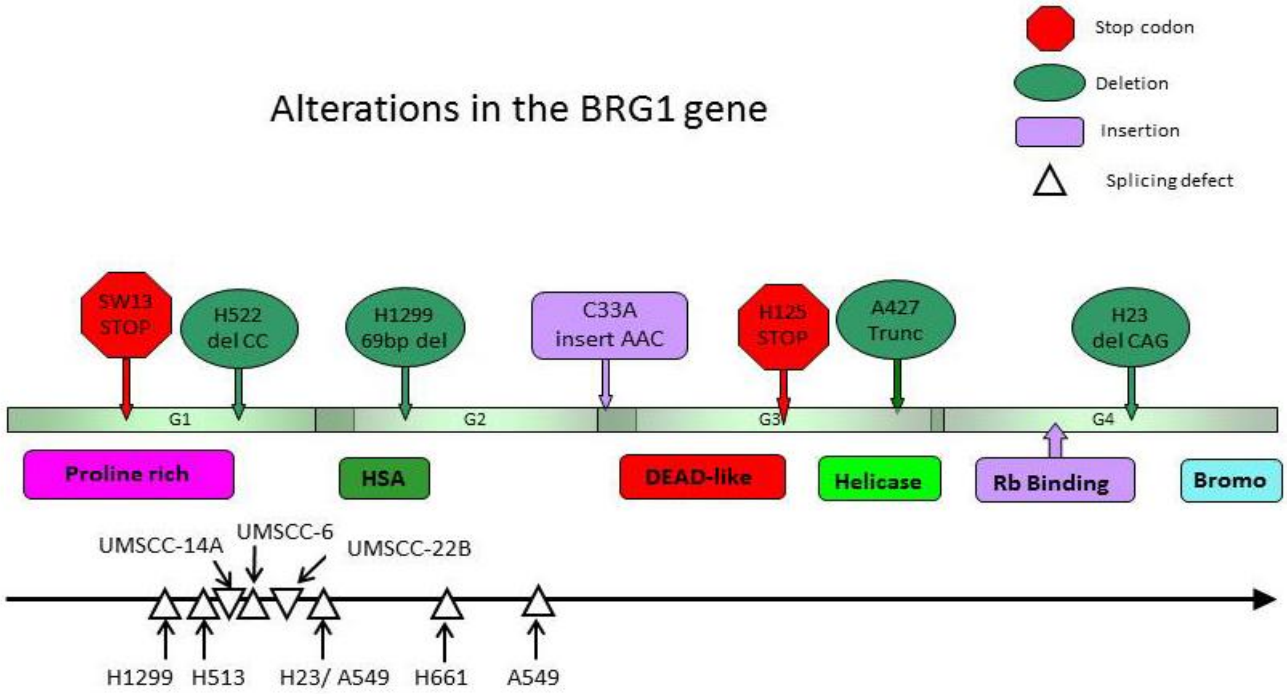

Supplementary Figure 2 ***Alterations in the BRG1 gene in cell lines.*** The cDNA from 17 cell lines was amplified by nested PCR. Alterations that were observed include stop codons, truncations, indels, mutations and splicing defects.

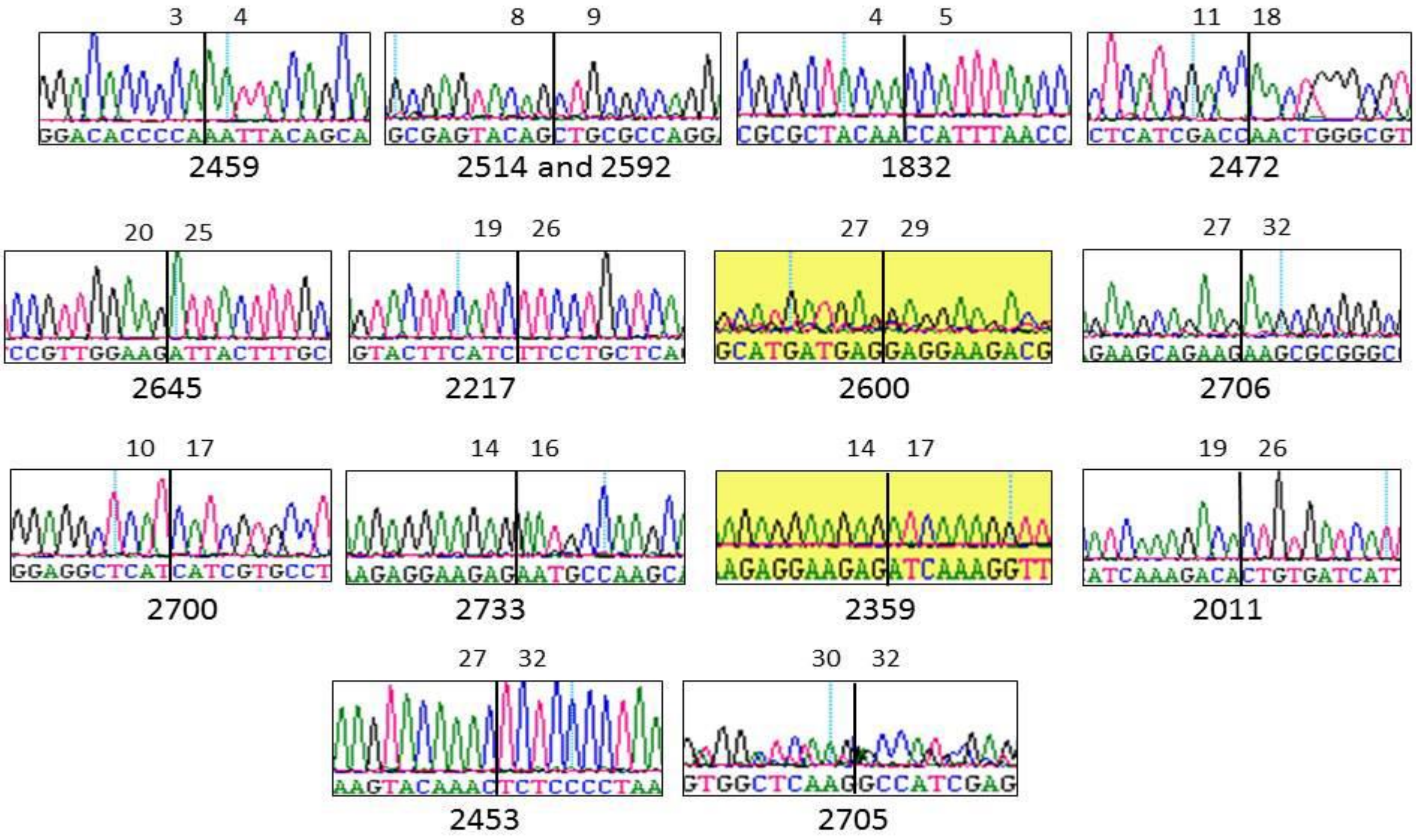

Supplementary Figure 3. ***BRG1-negative tumors have splicing defects.*** The cDNA from BRG1-negative tumors was amplified by nested PCR, and a total of 14 different splicing defects were observed in 15 different BRG1-negative tumors. The chromatograms for each tumor with alterations in splicing are illustrated. The numbers on either side of the combined chromatograms represent the exon numbers.

Primer	Amplicon Size (bp)	5' Forward	3' Reverse
3	448	ACTGGCTTCCTGTGGGATGT	AAGTAAGGCCCTCGGTCCAG
4	470	CTTGCCTTGGAGTCATG	ACGAGGTGGCCAGAACAGAG
5	569	GAGCTGGTGTAGGGGGAAGA	AACAGAGCACGGTCTGTCCA
6	448	GGACGCACTGTTTTCTCTTTTG	AGGATGGGTAATGAGTCCCACT
7	440	TCAGCAGCTTTCCATTTCCA	GCACAGGGCACTGTTTGCTA
8	437	GGCCAGCATCCTCTTCTGAG	GGCCGTAAGAGGAAGGCTCT
9	431	CCATACGTGTTTGTCATTGTGG	TCTTCTCCCTACCATTGGGTTT
10	313	GCCTTCTCTTTTGTGCTTTCCT	CCTCTAACTCCGAGCTGTGAAA
11/12	526	CCATGGGTGCTCACAGACAT	CACCCTGGGATTCTCACCAT
13/14	526	CCCACCTTGGCCTCTGTAAG	GCAGGAGACGGTCACAACAC
15	410	CCTCTCTGGGGGATGAACTG	CTCCCGCACACCTGACTGTA
16	487	CTTACAGTGCTGGCCACAGG	TTTCTGCTGTTGCACGTCCT
17	433	AGAGGGTGGGATGTGGCTTA	CCTTGACCCTGGACTTCTGG
18/19	875	TGATGAGAGACCGGCACTTG	TGTAGGGGCTTTGGAGGAGA
20	487	CTCTTCCACCTGGAGCCTTC	GCTCCATGCAGATGCAGACT
21	171	GGTAACGCTTGCTTCTCCTG	CTCCCCTCAAAACTCCATCA
22	430	TGGTCAGAGCTCATATGACAGG	AGCCAGTGTTTCATAGGCAAAG
23	467	AAAGCCACTCTTCCCCACTAGA	AGAGAAGTCTCCAAACGCTCCT
24	739	GGAGGAGTGTGAGGGAAAGG	GTGGTCAGCTCTCCAAGCAC
25	420	GGGAAATGGCTTTGCTGAAG	GAGGGAATGCCAGTGAGGAG
26	518	GCTGATCCTGCTCCTGCTCT	AAAAACCAAACCGACGGATG
27	409	CCACCTTCCCTTTTATGACCTC	TTTACTCCAAAGGGATGCAAAA
28/29	600	GGCTGAACGGAAAGAGGATG	GCAGAGTGGGATGCCTGAAC
30	492	CCAGGTCAGCCACTGAAAAA	CCCAAGGAAAGCATTTTGGA
31	248	GCGACAGAATGAGACCCTGT	CACCCACCACCAGAACTCTC
32	503	CCTTGAGAGTCCCAGTGTGTGT	AGTGTGCAGAGTCAGAGTGCAG
33/34	720	GACTCGGGGGTGATAGCC	AGCCCAGCTGTGGGAGTG
35/36	600	AGTCGGGCCCATCCACTC	AGCCTCAGCTTTCCCACCT
37cds	401	TCGATCTCCTGACCTCGTGA	GACAGGGCCAGTCACAAACA

Amplicon	Primer Name	Size (bp)	5' Forward	3'Reverse	
1A	0069+951	882	CTGTCTGCAGCTCCCGTGAAG	ATTCGCCATGGGTCCTTCAG	PCR
1A	0069+619	550	CTGTCTGCAGCTCCCGTGAAG	CTGAGCTGGTGCAGCTGGTT	PCR (nested)
1B	1403+834	569	TCATGGTATGGGAGGGCC	CTGCGCTTGTAGGCCTTAG	PCR
1B	1403+932	471	CTGAAGGACCCATGGCGA	CTGCGCTTGTAGGCCTTAG	PCR (nested)
2	1268+2613	1345	GCGAACCAAAGCGACCATTGAG	GACAAAGGCCCGTCTTGCTG	PCR
2	1299+2594	1295	CAGGCTGCTGAACTTCCAGA	GCTGGGGATCCCTTGTAAGA	PCR (nested)
3	2499+3680	1181	CATCATCGTGCCTCTCTCAAC	ACACGCACCTCGTTCTGCTG	PCR
3	2529+3635	1106	CTGGGCGTACGAGTTTGACA	GCTTGCAGGTCCTGGTGAG	PCR (nested)
4	3568+4638	1070	AACCTCCAGTCGGCAGACAC	GCGGTACTTGTGGTTGCGAA	PCR
4	3568+4453	885	AACCTCCAGTCGGCAGACAC	TGAGGTTGGGTGGGTTAGGG	PCR (nested)

Supplementary Table 1A and 1B *PCR Primer sequences.* Table 1A: All 37 exons of BRG1 were amplified from the genomic DNA of the BRG1-negative tumors by PCR. In the case of BRG1, exon 3 according to our number system corresponds to the start codon. Table 1B: cDNA from the BRG1-negative tumors was amplified by nested PCR using 5 sets of overlapping primers that correspond to amplicons 1A, 1B, 2, 3 and 4.

Supplementary Table 2A: Staining Product

	"0-40"	"41-100"	"101-200"	"201-300"	Total	Percent Negative
Kidney Cancer	27	14	6	2	49	55
Bladder Cancer	16	28	52	39	135	12
Breast Cancer	12	20	6	0	38	32
Cholangiocarcinoma	15	7	8	1	31	48
Colon Cancer	40	28	13	2	83	48
GIST	20	12	11	2	45	44
Liver Cancer	36	18	4	2	60	60
Lung Cancer	41	49	112	58	260	16
Melanoma	5	7	27	11	50	10
Ovarian Cancer	47	73	91	41	252	19
Thyroid Cancer	3	11	16	8	38	8
Head/neck Cancer	13	7	14	38	72	18
Pancreatic Cancer	4	5	17	8	34	12
Stomach Cancer	0	1	46	38	85	0
Cervical Cancer	6	1	6	15	28	21
Esophageal Cancer	2	6	21	38	67	3
Prostate Cancer	1	28	20	1	50	2
Brain Cancer	16	8	3	5	32	50

Supplementary Table 2B: Staining Product

Bronchioloalveolar Carcinoma	"0-40"	"41-100"	"101-200"	"201-300"	Total	Percent Negative
Mucinous	0	8	37	12	57	0.0
Non-mucinous	4	4	44	64	116	3.4
All	4	12	81	76	173	2.3

Supplementary Table 2C: Intensity of Staining 0= none 3= most intense

NeuroEndocrine Lung Cancer	"0-40"	"41-100"	"101-200"	"201-300"	Total	Percent Negative
Carcinoid	1	12	18	2	33	3.0
Atypical Carcinoid	0	3	2	0	5	0.0
Small Cell	2	8	51	27	88	2.0
Large Cell	2	7	15	4	28	7.0
All	5	30	86	33	154	3.0

Supplementary Table 2D Intensity of Staining 0= none 3= most intense

Brain	"0-40"	"10-100"	"101-200"	"201-300"	Total	Percent Negative
Glioblastoma	9	4	2	3	18	50.0
Meningioma	3	2	0	0	5	60.0
Astrocytoma	4	2	1	2	9	44.0
All	16	8	3	5	32	50.0

Supplementary Table 2E: Intensity of Staining 0= none 3= most intense						
Cervical	“0-40"	“41-100"	“101-200"	“201-300"	Total	Percent Negative
CIN1	1	12	0	0	13	7.7
CIN2/3	0	3	6	1	10	0.0
Squamous	3	1	6	14	24	12.5
Adenocarcinoma	3	0	0	1	4	75.0
All	7	16	12	16	51	13.7

Supplementary Table 2F: Intensity of Staining 0= none 3= most intense						
Esophageal	"0-41"	“41-100"	“101-200"	“201-300"	Total	Percent Negative
Barrett's Esophagus	7	0	4	5	16	43.7
Barrett's Dysplasia	3	0	7	5	15	20.0
Esophageal Cancer	2	6	21	38	67	3.0
All	12	6	32	48	98	12.2

Supplementary Table 2G: Intensity of Staining 0= none 3= most intense						
Prostate Cancer	“0-40"	“41-100"	“101-200"	“201-300"	Total	Percent Negative
Benign	3	21	4	0	28	11.0
PIN	0	20	6	0	26	0.0
Cancer, Gleason 3-5	0	15	13	1	29	0.0
Mets	1	13	7	0	21	5.0

Supplementary Table 2A-G. *BRG1 Loss in Selected Cancers.* Supplementary Table 2A: a compilation of the 18 cancer types that are featured in Figure 2 is shown. Tumors that were considered negative (product score 0-40) were summed and the percentage of BRG1-negative tumors was calculated for each cancer type. Supplementary Table 2B-D: the percentage of BRG1-negative tumors of several histologic subtypes of lung, ovarian and brain cancer were calculated. Supplementary Tables 2E-G: the percentage of BRG1-negative premalignant lesions and malignant tumors for cervical cancer, esophageal cancer and prostate cancer were tabulated. All scoring was performed on the respective TMAs according to the system described in the Materials and Methods.
